# Management of Metastatic Renal Cell Carcinoma Following First-Line Immune Checkpoint Therapy Failure: A Systematic Review

**DOI:** 10.3390/cancers16142598

**Published:** 2024-07-20

**Authors:** Fausto Petrelli, Ivano Vavassori, Mauro Rossitto, Lorenzo Dottorini

**Affiliations:** 1 Oncology Unit, ASST Bergamo Ovest, 24047 Treviglio, BG, Italy; 2 Urology Unit, ASST Bergamo Ovest, 24047 Treviglio, BG, Italy

**Keywords:** renal cell carcinoma, first line, immune checkpoint inhibitors, second-line therapy, systematic review

## Abstract

**Simple Summary:**

Metastatic renal cell carcinoma (RCC) remains a challenging cancer to treat, especially when patients do not respond to initial immune checkpoint inhibitor (ICI) therapy. This systematic review aims to identify effective second-line treatments for patients who have failed first-line ICI therapy. By analyzing 27 studies involving 1970 patients, we found that both VEGFR tyrosine kinase inhibitors (TKIs) and ICIs can be beneficial as second-line treatments, with VEGFR TKIs showing slightly higher response rates. The findings from this research could guide oncologists in personalizing treatment strategies, ultimately improving outcomes for patients with metastatic RCC.

**Abstract:**

Introduction: There is a significant gap in the literature concerning the effective management of second-line therapy for patients with metastatic renal cell carcinoma (RCC) who have received immune checkpoint inhibitors (ICIs). Most of the published articles were small multicenter series or phase 2 studies. To our knowledge, a systematic review that comprehensively outlines the range of treatment options available for patients with metastatic RCC who do not respond to first-line ICIs has not yet been conducted. Our aim was to synthesize evidence on second-line therapies for patients with metastatic RCC after initial treatment with ICIs and to offer recommendations on the best treatment regimens based on the current literature. Material and Methods: We conducted a search in PubMed, Embase, and the Cochrane Library on 29 February 2024, following the Preferred Reporting Items for Systematic Reviews and Meta-Analyses (PRISMA) guidelines. We selected articles that met the predetermined inclusion criteria (written in English, retrospective observational studies, prospective series, and randomized trials reporting second-line therapy for metastatic RCC after failure of ICI-based therapy). Relevant articles were identified in the reference lists. The main endpoint was the overall response rate (ORR), with the median progression-free survival (PFS) and overall survival (OS) as secondary endpoints. Results: We included 27 studies reporting the outcomes of 1970 patients. Salvage therapies were classified as targeted therapy (VEGFR TKIs) in 18 studies and ICIs in 8 studies. In studies where TKIs were the second line of choice, the pooled ORR was 34% (95% CI: 30.2–38%). In studies where ICIs, alone or in combination with TKIs, were used as second-line therapies, the ORR was 25.7% (95% CI: 15.7–39.2%). In studies where TKIs and ICIs were the second-line choices, the pooled median PFS values were 11.4 months (95% CI: 9.5–13.6 months) and 9.8 months (95% CI: 7.5–12.7 months), respectively. Conclusions: This systematic review shows that VEGFR TKIs and ICIs are effective second-line therapies following an initial treatment with anti-PD(L)1 alone or in combination. The treatment choice should be personalized, taking into account the patient’s response to first-line ICIs, the site of the disease, the type of first-line combination (with or without VEGFR TKIs), and the patient’s overall condition.

## 1. Introduction 

The management of metastatic clear cell renal cell carcinoma (RCC) has undergone significant advancements in the past two decades, primarily due to the introduction and widespread use of immune checkpoint inhibitors (ICIs) as a foundational element of first-line treatment. These advancements have greatly impacted the treatment landscape, providing new hope and improved outcomes for patients with aggressive cancer. ICIs, which inhibit proteins that hinder the immune system from attacking cancer cells, have revolutionized the therapeutic approach, resulting in enhanced survival rates and improved quality of life for many patients. However, a substantial number of patients either do not respond adequately to first-line ICI therapy or experience disease progression, posing a complex clinical challenge that requires additional therapeutic interventions. The variability in response to ICIs highlights the need for personalized treatment strategies and underscores the complex nature of metastatic RCC. The mechanisms driving resistance and progression after ICI therapy remain not fully understood, further complicating treatment decision making. Historically, for patients exhibiting progression following standard first-line therapy, the use of other vascular endothelial growth factor (VEGF) tyrosine kinase inhibitors (TKIs), such as cabozantinib (CABO), or mammalian target of rapamycin (mTOR) inhibitors, such as everolimus, has been common. These agents have demonstrated efficacy in the second-line setting and beyond, presenting crucial options for patients who fail ICI therapy. Network meta-analyses have shown that CABO and nivolumab (NIVO) outperform everolimus, demonstrating an efficacy comparable to that of axitinib [1]. These findings reinforce the role of TKIs and mTOR inhibitors as vital components in the therapeutic arsenal against metastatic RCC. With the integration of ICIs into the therapeutic framework for metastatic RCC, strategies for managing patients who do not respond or progress after ICI therapy have continued to evolve. These strategies include standard TKIs, ICI rechallenge, or novel targeted therapies. The dynamic and evolving nature of treatment protocols necessitates ongoing research and the adaptation of clinical practices to ensure optimal patient outcomes. Exploring and refining these second-line treatment options are crucial to provide the best possible care to patients who do not benefit from an initial ICI therapy. Addressing the current knowledge gaps, suggesting future research directions, and providing essential insights and recommendations for clinicians are imperative to navigate the complex decision-making process in treating metastatic RCC after initial ICI therapy failure. Understanding the mechanisms of resistance and identifying biomarkers predictive of response to subsequent therapies are critical areas of ongoing research. We conducted a systematic review to consolidate the existing evidence and analyze the therapeutic approaches employed in managing metastatic RCC after the failure of first-line ICIs. 

## 2. Materials and Methods

This systematic review and meta-analysis adhered to the Preferred Reporting Items for Systematic Reviews and Meta-Analyses (PRISMA) guidelines. Notably, this investigation was not conducted in this study. The protocol for the systematic review of our study was not registered for PROSPERO.

### 2.1. Search Strategy and Selection Criteria

We conducted a comprehensive literature search up to February 29, 2024, using the PubMed, Embase, and Cochrane Library databases. The search strategy employed a combination of medical subject heading (MeSH) terms and keywords as follows: (“renal cell carcinoma” or “RCC”) AND (PD-1 or PD-L1 or “immune checkpoint inhibitors”) AND (second-line or pretreated or progress* or failure or “previously treated”). No restrictions were placed on language, geographical region, patient age, or follow-up duration.

#### 2.1.1. Inclusion Criteria (PICO Criteria)

1. Prospective or retrospective clinical studies.
2. Patients with any histological type of RCC undergoing treatment with PD-1/PD-L1 inhibitors alone or in combination with antiangiogenic or anti-CTLA4 agents as initial therapy.
3. Reports on outcomes such as progression-free survival (PFS), overall survival (OS), overall response rate (ORR), and stable disease rates after second-line treatment.

#### 2.1.2. Exclusion Criteria

1. Editorials, letters, reviews, or case reports.
2. In vitro or animal research.
3. Studies without initial treatment involving ICIs.
4. Lack of relevant outcome reporting.
5. Treatments beyond the third-line.
6. Replicated publications.

### 2.2. Quality Appraisal and Data Extraction

Data extraction was independently performed by two analysts (FP and MR), and any disagreements were resolved by consulting a third expert (LD). Extracted data included study specifics, such as authorship, publication year, study design, median follow-up, disease histology, treatments, therapy line, cohort size, primary outcome (ORR), and secondary endpoints (stable disease rate, median PFS, and OS). Two authors (FP and AS) independently assessed the risk of bias using the RoBINS tool. Any disagreements were resolved through discussion and arbitration with a third senior author (AL) if needed. We evaluated the methodological quality of the observational studies using the Newcastle–Ottawa Scale (NOS), a tool commonly used in evidence-based healthcare to evaluate the quality of non-randomized studies, especially cohort and case–control studies. A score of at least 7 indicates higher-quality evidence and a lower risk of bias, whereas lower scores suggested moderate-to-poor quality studies.

### 2.3. Statistical Analysis

The synthesis of ORR and stable disease rates, median PFS, and OS metrics was conducted using Comprehensive Meta-Analysis version 4.0.000. In addition to subgroup assessments, the analysis considered potential sources of heterogeneity. Inter-study variability was quantified using Cochran’s Q and I^2^ indices, with Q’s significance set at below 0.1 and I^2^ exceeding 50%, indicating substantial heterogeneity. Predominance was placed on I^2^ in cases of discrepancy, acknowledging Q’s restricted sensitivity in detecting heterogeneity. Analytical models were selected based on the degree of heterogeneity: fixed effects for low variation and random effects for significant variation.

## 3. Results

### 3.1. Study Selection and Characteristics 

**Figure 1:**
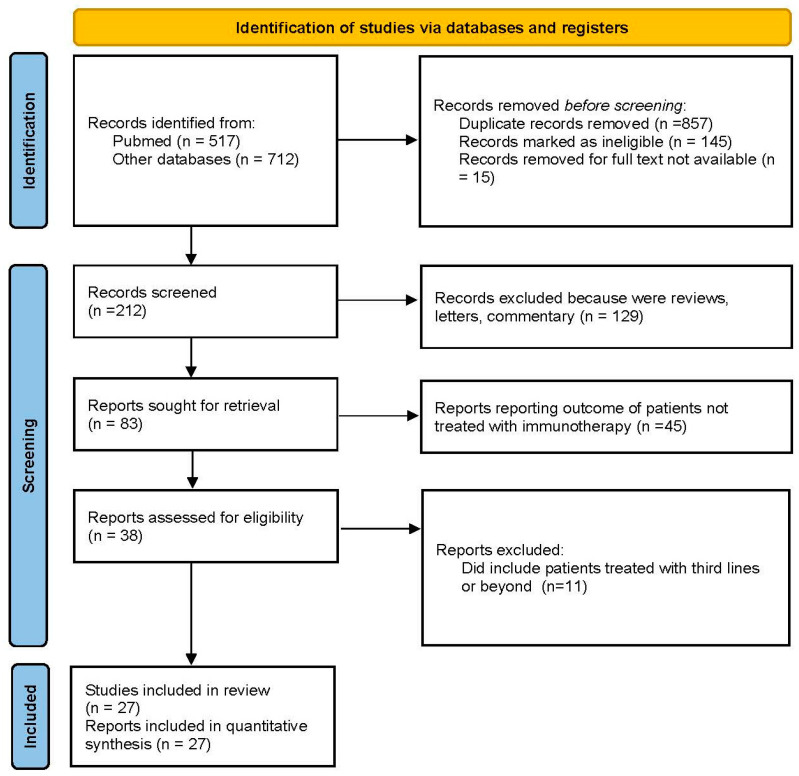
Flow diagram of the included studies.

As depicted in Figure 1, 1229 studies were identified through the database searches. After removing duplicates, screening by title and abstract, and conducting a detailed assessment of potentially relevant studies, 27 studies were selected for inclusion in this meta-analysis, involving 1970 patients [2,3,4,5,6,7,8,9,10,11,12,13,14,15,16,17,18,19,20,21,22,23,24,25,26,27,28]. Table 1 presents the baseline characteristics and primary outcomes of the included studies. Among these, 7 were phase 2 studies, 1 was a phase 3 study, 2 were prospective series, and 17 were retrospective studies. The median follow-up period ranged from 3.7 to 49.9 months.

### 3.2. VEGFR Tyrosine Kinase Inhibitor Studies

Eighteen studies involving 1,310 patients evaluated various VEGFR TKIs as second-line or subsequent therapies. Cabozantinib (CABO) was the most frequently studied TKI, featured in six studies either alone or in combination with belzutifan in one study. Other VEGFR TKIs included sunitinib, axitinib, and pazopanib, used either alone or in various combinations. The proportion of patients receiving second-line TKI therapy ranged widely from 2.8% to 100%, with a median of 100%. This variation reflects the different clinical practices and patient selection criteria across the included studies.

### 3.3. Immune Checkpoint Inhibitor (ICI) Studies

Eight studies, encompassing 594 patients, investigated the use of ICIs, either alone or in combination with other treatments, as second-line therapy. Four studies explored ICIs alone or in combination with ipilimumab (IPI), one study used atezolizumab plus bevacizumab, two studies examined pembrolizumab in combination with lenvatinib or axitinib, and one phase 3 trial evaluated atezolizumab plus cabozantinib versus cabozantinib alone. The diversity of ICI regimens reflects ongoing efforts to optimize immunotherapy strategies in metastatic RCC.

### 3.4. Quality Assessment and Risk of Bias

The quality assessment using the Newcastle–Ottawa Scale (NOS) indicated that 15 studies were of moderate to high quality, while 11 were considered of poor quality. The risk of bias, evaluated using the ROBINS-I tool, indicated that 5 studies had a high risk of bias, 5 had a moderate risk, and 17 had a low risk. The main sources of bias included retrospective study designs, heterogeneous patient populations, and varying follow-up durations. Despite these limitations, the included studies provided valuable insights into the efficacy of second-line treatments in metastatic RCC.

### 3.5. Overall Response Rate (ORR) and Stable Disease (SD)

**Figure 2:**
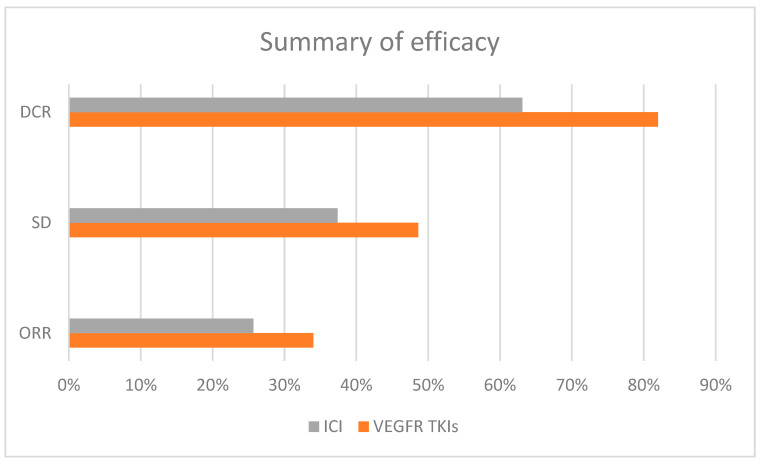
Summary of efficacy of second-line studies (ORR, SD, and DCR rates) for VEGFR TKIs and ICIs (ORR, overall response rate; SD, stable disease rate; DCR, disease control rate).

In studies where VEGFR TKIs were used as the second-line treatment, the pooled ORR was 34% (95% CI: 30.2–38%), and the rate of stable disease was 48.6% (95% CI: 43.2–53.8%), resulting in an overall disease control rate of 82% (Figure 2). In contrast, studies using ICIs alone or in combination with TKIs as second-line therapies reported a pooled ORR of 25.7% (95% CI: 15.7–39.2%) and a stable disease rate of 37.4% (95% CI: 28.4–47.3%), resulting in an overall disease control rate of 63.1%. A meta-regression analysis showed that the effect size (ORR) was not significantly associated with prior exposure to TKIs during first-line treatment (*p* = 0.8). These findings suggest that, while VEGFR TKIs may achieve higher response rates, ICIs also provide substantial clinical benefit in the second-line setting.

### 3.6. Progression-Free Survival (PFS) and Overall Survival (OS)

For studies utilizing VEGFR TKIs as the second-line treatment option (*n* = 13), the pooled median PFS was 11.4 months (95% CI: 9.5–13.6), and the median OS was 16.2 months (*n* = 9; 95% CI: 13.2–19.8). For studies involving ICIs as second-line therapies (*n* = 6), the pooled median PFS was 9.8 months (95% CI: 7.5–12.7). The overall survival data were limited, reported in only three studies, with median OS ranging from 23.8 to 25.7 months. These results indicate that VEGFR TKIs provide a modest advantage in delaying disease progression compared to ICIs, although both treatment modalities offer comparable overall survival benefits.

### 3.7. Publication Bias

Based on the extracted data, we present a summary of the Risk of Bias (RoB) and the Newcastle–Ottawa Scale (NOS) scores for the included studies:

The Newcastle–Ottawa Scale (NOS) was used to evaluate the methodological quality of the included studies, particularly the observational ones. The NOS score ranges from 0 to 9, with a score of 7 or higher indicating high-quality studies.

High Quality (NOS score ≥ 7): 15 studies.

Moderate Quality (NOS score = 6): 11 studies.

Poor Quality (NOS score < 6): 1 study.

The Risk of Bias (RoB) was assessed using the Risk of Bias in Non-randomized Studies of Interventions (ROBINS-I) tool. The studies were categorized into three levels based on their risk of bias:

Low Risk of Bias: 17 studies.

Moderate Risk of Bias: 5 studies.

High Risk of Bias: 5 studies.

## 4. Discussion

The treatment landscape of RCC has evolved significantly in recent years. However, not all patients respond to first-line therapies, which poses a challenge for subsequent treatment strategies. The management of metastatic RCC after first-line therapy failure presents a rapidly developing field enriched with multiple second-line treatment options. The choice of second-line therapy depends on various factors, including the type of first-line treatment, the patient’s clinical profile, the outcomes and side effects of prior treatments, and prognostic evaluations. Many patients with RCC require second-line therapy due to disease progression or adverse effects from initial treatment. Notably, after first-line therapy with a VEGFR TKI, novel agents such as CABO and NIVO are commonly recommended. With the advent of ICIs, either as monotherapy or in combination with antiangiogenic agents, a new standard for the first-line treatment of metastatic RCC has been established. With progress in ICIs, various second-line therapies have shown promise. Nevertheless, research continues to refine these strategies, with current insights primarily derived from limited randomized studies. In the present and future scenarios, various strategies are emerging for the treatment of ICI failure. Firstly, combination therapies involve the use of ICIs in conjunction with other therapeutic modalities, such as TKIs, to enhance the antitumor efficacy. For example, the addition of VEGF inhibitors can normalize the tumor vasculature, thereby improving immune cell infiltration and enhancing the effectiveness of ICIs. Secondly, the development of biomarkers is important for identifying predictive factors associated with ICI response, which helps in tailoring treatments to individual patients. Biomarkers such as PD-L1 expression, tumor mutational burden (TMB), and specific genetic mutations can guide the selection of appropriate therapies. Thirdly, targeting the tumor microenvironment (TME) is a potential strategy for reducing immunosuppression and enhancing immune activation. This can involve the targeting of immunosuppressive cells or cytokines within the TME. Lastly, microbiome modulation through interventions like probiotics or fecal microbiota transplantation holds promise in improving ICI responses by restoring a healthy gut microbiome [29].

Our comprehensive review systematically analyzed the literature on the topic, focusing on patients who were administered second-line agents after first-line ICI failure. We observed that second- or further-line therapies involving TKIs and ICIs resulted in an ORR of 25–30% and a stable disease rate of 50–60%. The median PFS ranged from 10 to 11 months, while the median OS was often unreported because it was not reached or unavailable, although it spanned from 16 months with TKIs to 24 months with ICIs.

The median PFS and OS also vary across studies, which reflects the heterogeneity in patient populations, treatment regimens, and follow-up durations. Some studies report that the PFS and OS were not reached within the study period, suggesting prolonged disease control and survival in certain patient cohorts. It is important to recognize these patients, as well as those with rapid disease progression that warrant further research efforts. In more detail, several studies have investigated the combination of NIVO and IPI, showing variable efficacy. While the combination tends to have a higher response rate, it is also associated with potential immune-related adverse events. The use of TKIs after ICI therapy has demonstrated effectiveness in the second-line setting, but the success varies, highlighting the need for personalized treatment approaches. Furthermore, recent studies explore combinations such as pembrolizumab with lenvatinib or belzutifan with CABO, suggesting promising avenues for future research and potential improvements in patient outcomes.

In the era before immunotherapy, NIVO or CABO were the main choices for patients who had progression on their initial anti-angiogenic agent treatment. The phase III METEOR trial and the CheckMate 025 trial demonstrated superior outcomes with these treatments compared to everolimus, showing improvements in ORR, PFS, and OS. Additionally, the combination of lenvatinib with everolimus presents a viable option for patients with clear cell RCC who are progressing after antiangiogenic therapy. This is supported by improved PFS over everolimus alone in randomized trials [30,31,32].

Our findings provide three critical insights. First, our data encapsulate and surpass the historical figures from the pre-immunotherapy era, indicating a significant improvement in ORR and PFS in our patient cohort, most of whom were previously treated with ICIs alone or with anti-CTLA-4 agents. Second, no single TKI emerged as preferable, with a wide variation in associated antitumor activity. The pooled ORR exceeded 30% in the CABO and non-CABO studies. Lastly, despite the negative outcomes of the CONTACT-03 study (CABO + atezolizumab vs. CABO alone), the combinations of ICI with IPI or TKIs have demonstrated a disease control rate of 63%, even after exposure to a single-agent ICI. Adding IPI to NIVO may enhance response rates following progression to single-agent NIVO, as evidenced in the TITAN-RCC study and other analyses included in our review [33,34].

Second-line and subsequent therapies are crucial for extending OS from the initiation of the first-line treatment throughout the course of the disease. When the median PFS with first-line ICIs ranges from 10 to 20 months and the median OS exceeds 3–4 years, post-progression survival (from the start of second-line treatment until death) may account for more than 50% of the total lifespan. Sequential treatment selection is crucial during disease progression, reserving later options for palliative care, which are usually linked to only a few months of PFS (e.g., everolimus). After second-line TKIs, it is common to consider rechallenging with an mTOR inhibitor or ICI.

The first step was the approval of CABO, NIVO, and lenvatinib for patients who experienced disease progression after antiangiogenic therapy. This marked a significant step forward, demonstrating improved OS compared to everolimus. However, at least four preferred combinations are available as first-line therapies: three are available for every clinical risk disease (pembrolizumab + axitinib, lenvatinib, or CABO + NIVO) and one for poor/intermediate-risk RCC (NIVO + IPI). For patients commencing treatment with a combination of ICI + axitinib or lenvatinib, CABO may be indicated as a second-line option, whereas for patients starting with CABO + NIVO therapy, alternative TKIs are available (sunitinib, axitinib, or pazopanib). Finally, for those with poor/intermediate-risk disease starting with NIVO + IPI, all available TKIs (CABO, sunitinib, axitinib, or pazopanib) are viable options, despite the fact that, in the METEOR study, OS improvement vs. everolimus in poor-risk disease was not significant [35]. Patients requiring second-line therapy for mRCC were heterogeneous, with most having a good or intermediate prognostic profile and multiple metastatic sites, emphasizing the need for tailored second-line treatment strategies [36].

Rechallenging with ICIs in RCC has also shown promise and is another option. However, the efficacy and outcomes can depend on patient-specific factors and the nature of the progression. In particular, the study conducted by Pal et al. in 2023 as part of the CONTACT-03 trial aimed to examine the effectiveness and safety of combining atezolizumab with CABO in metastatic RCC patients who had previously experienced progression on ICIs [33]. Regrettably, the study found that this combination did not yield significant improvements in clinical outcomes when compared to CABO monotherapy. Specifically, the median PFS was 10.6 months for the combination therapy group and 10.8 months for the monotherapy group, indicating no substantial difference. Similarly, the median OS was 25.7 months for the combination therapy compared to an unevaluable outcome for CABO alone. Both PFS and OS results did not exhibit statistically significant improvements with the addition of atezolizumab to CABO alone, suggesting that the efficacy of current second-line therapies has reached a plateau. Furthermore, the study observed an increase in toxicity within the combination therapy group. Common adverse events included diarrhea, decreased appetite, and hypothyroidism. The combination therapy group also experienced a higher incidence of serious adverse events and treatment discontinuations. These findings indicate that, although the rationale behind combining atezolizumab with CABO was based on their potential complementary mechanisms, this combination did not enhance clinical outcomes in this specific patient population. In a retrospective study by Ravi et al., 69 patients were included, with the most prevalent therapies being single-agent ICI or dual ICIs. The ORR at rechallenge was 23%, which is consistent with our findings. The probability of a response to rechallenge was the highest among patients who had previously responded to ICI treatment. Nonetheless, responses were also observed in patients who had progressive disease as their best outcome following first-line ICI therapy as well as in those who underwent single-agent ICI rechallenge. In a pooled analysis of four studies involving rechallenge with NIVO + IPI after prior anti-PD-1/PD-L1 therapy failure, the pooled ORR was 10.0%, while the incidence of grade ≥ 3 immune-related adverse events was 27.0% [37,38]. Consequently, these studies emphasize the need for further research and the careful consideration of sequential immune checkpoint inhibitor use in RCC.

The potential limitations of this review deserve acknowledgment. First, the reviewed studies often involved a wide range of second-line therapies, leading to variability in the outcomes. The diversity in this context can obscure clear conclusions and highlight the challenges of comparing various treatment modalities. Many of the included patients were identified from a retrospective database series and lacked prospective selection or enrollment in phase 3 trials. This methodological approach may have introduced bias and limited the generalizability of the findings. Second, our review included a series of patients who were treated as third or subsequent lines of therapy and often did not immediately transition to treatment following a prior course of upfront ICI therapy. In addition, it is important to note that some patients receiving second-line ICI therapy might have discontinued first-line ICI therapy for reasons that have not been fully documented. The outcomes of subsequent ICI treatments, such as toxicity or initial response, could be influenced by the reasons for discontinuation, potentially skewing the effectiveness of subsequent therapies. Moreover, there is a notable variation in the initial ICI regimens, with many patients receiving monotherapy (e.g., anti-PD(L)1 alone or in combination with IPI). This variation could affect the comparative effectiveness of TKIs versus historical controls, suggesting that initial treatment choices influence subsequent therapy outcomes. Finally, the diversity of treatments across studies and the limited number of studies per intervention type constrained our ability to conduct the meta-analyses. This limitation is compounded by incomplete information from various publications, making it challenging to draw firm conclusions regarding the efficacy of specific second-line therapies.

Salvage therapies for RCC are evolving, with a shift towards personalized treatment approaches based on patient characteristics, biomarker profiles, and responses to prior treatments. The integration of targeted therapies, immunotherapies, and novel agents offers hope for improved outcomes in salvage settings. Ongoing clinical trials are exploring new drugs and combinations, including novel TKIs, immunotherapies, and agents targeting other pathways, such as HIF-2α [39]. Identifying biomarkers that predict responses to specific therapies could also enable more personalized approaches to salvage therapy. Moreover, strategies aimed at modifying the tumor microenvironment to enhance the efficacy of immunotherapies are under investigation.

The selection of second-line therapy for RCC following first-line treatment with ICIs is a pivotal decision influenced by the efficacy of prior treatment, patient performance status, and tumor characteristics, and significantly influences the subsequent course of the disease. The analyzed studies highlight the evolving landscape of RCC treatment, especially following first-line ICI therapy. It is crucial to consult current guidelines and clinical trial data and consider patient-specific factors when choosing second-line treatment to achieve the best possible outcome for patients with RCC. Drawing on data from both small phase 2 trials and real-world observational studies, patients undergoing salvage therapy received a variety of treatments, predominantly VEGFR TKIs or ICI rechallenges, showing comparable efficacy.

Generally, conventional second-line TKIs, such as CABO, are favored after first-line treatments that do not include CABO. On the other hand, following the administration of NIVO plus CABO, axitinib, sunitinib, or pazopanib may be selected. After combinations of NIVO and IPI, any TKI can be chosen, and there is the possibility of reintroducing an ICI after disease control, provided discontinuation is necessary due to toxicity or other clinical reasons. Future research is of paramount importance in order to accurately define the characteristics and subtypes of patients who may benefit from personalized approaches to second-line treatment.

The choice of second-line therapy should be personalized, considering factors such as: (1) Patient response to first-line ICI: Patients’ responses to initial ICI therapy can guide the selection of second-line treatments. Those who initially responded to ICIs may benefit from ICI rechallenge or combination therapies. (2) Disease characteristics: The type and location of metastases, as well as specific tumor characteristics, should inform treatment decisions. (3) Patient overall condition: Performance status, the presence of comorbidities, and other individual factors should be considered to optimize treatment outcomes.

In conclusion, this systematic review highlights that both VEGFR tyrosine kinase inhibitors (TKIs) and immune checkpoint inhibitors (ICIs) are effective second-line therapies for patients with metastatic renal cell carcinoma (RCC) who have experienced failure with initial ICI treatments. Our analysis of 27 studies involving 1970 patients shows that VEGFR TKIs provide a slightly higher overall response rate (ORR) compared to ICIs, making them a valuable option for second-line treatment. However, the effectiveness of these therapies can vary based on individual patient characteristics and the specifics of their disease progression.

The personalization of treatment is crucial. The decision on second-line therapy should consider the patient’s response to first-line ICIs, the location of metastases, the type of first-line combination therapy used, and the patient’s overall condition. The heterogeneous nature of RCC and the varying responses to treatment underscore the need for tailored therapeutic approaches. Future research should focus on elucidating the mechanisms of resistance to ICIs and identifying predictive biomarkers for treatment responses. Such advancements will enable more precise and effective management strategies for patients with metastatic RCC, ultimately improving clinical outcomes.

## Figures and Tables

**Table 1:** Summary of the reviewed studies, including study characteristics, patient demographics, interventions, and key outcomes.

Author/Year	Type of Study/Median Follow-Up (Months)	Country	N° pts	Clear Cell RCC %	First-Line Type (%)	Second-Line Type (%)	Second Line (%)	Further Lines (%)	ORR (%)	SD (%)	Median PFS (Months)	Median OS (Months)	NOS Score (Quality)/RoB
Atkins/2022 [4]	Phase 2/26.9	US	35	100	NIVO (100)	NIVO + IPI	100	0	11.4	25.7	-	Not reached	8/low
Auvray/2019 [5]	Retrospective/8	France	33	100	NIVO + IPI (100)	AXI (24.2), CABO (6.1), PAZO (18.2), SUN (51.5)	100	0	36	39	8	13	6/moderate
Barata/2018 [2]	Retrospective/6.4	UK	33	100	ATEZO + BEV (64), NIVO + IPI (33)	AXI (48), CABO (12), PAZO (27), SUN (12)	100	0	29	54	6.4	-	6/moderate
Buchbinder/2019 [3]	Retrospective/8.6	US	17	-	ICI (100)	HD IL2	100	0	24	47	8.6	-	6/high
Cao/2022 [7]	Retrospective/5.1	US	182	100	NIVO or NIVO + IPI (82), PEMBRO or IPI (15)	PAZO	100	0	-	-	16	Not reached	6/low
Graham/2021 [12]	Retrospective/NR	US	104^^	85.3	NIVO or NIVO + IPI (100)	CABO (27), SUN (34), PAZO (37), other (1), mTORi (1)	100	0	29.8	NR	-	-	5/low
Grande/2022 [11]	Phase 2/15	Spain	21	100	ICI (86) **, ICI + TKI (14)	SUN	100	0	19	67	5.6	23.5	5/high
Gul/2020 [10]	Retrospective/12	US	23 ##	89	ICI (100)	NIVO + IPI	100	0	13	26	-	-	7/low
Kato/2021 [21]	Retrospective/NR	Japan	38	81.6	NIVO or NIVO + IPI (100)	AXI (84.2), CABO (5.3), PAZO (5.3), SUN (5.3)	100	0	42.1	28.9	-	-	6/high
Pal/2023 [14]	Phase 3/15.2	Internat.	522	78	NIVO + IPI (54), PEMBRO + AXI (46)	ATEZO + CABO VS CABO	100	0	41/41	51/48	9.9/10.3 ^^	25.7/not reached	-/low
Powles/2022 [19]	Prospective/19.4	US	44	91	ICI (ATEZO)	ATEZO + BEV	100	0	25	-	11.1	-	7/moderate
Procopio/2023 [22]	Phase 2/11.9	Italy	31	87	ICI (63) or ICI + TKI (37)	CABO	100	0	27	43	8.3	13.8	7/low
Santoni/2022 [20]	Retrospective/25.7	Italy	57	77	ICI (68) or ICI + TKI (32) °	CABO	100	0	21	25	6.9	8.84	8/low
Shah/2019 [23]	Retrospective/14.9	US	70	100	ICI (64) or ICI + BEV (36)	PAZO (27), SUN (9), AXI (36), CABO (28)	100	0	41.2	52.9	13.2	Not reached	7/low
Tomita/2021 [25]	Retrospective/20.3	Japan	19	-	NIVO + IPI	AXI or SUN (79)	100	0	32	53	32	Not reached	7/low
Fitzgerald/2023 [9]	Retrospective/32	US	107	100	ICI (52) or ICI + TKI (48)	CABO (38), AXI (15), PAZO (5), SUN (3), ICI (13), ICI + TKI (13)	61.8	38.2	33	58	-	-	8/low
McGregor/2020 [16]	Retrospective/12	US	86	100	ICI (71), ICI-TKI (29)	CABO	60.5	39.5	36	43	6.5	13.1	7/low
Hahn/2023 [13]	Retrospective/49.9	US	57	100 #	ICI (73.7), ICI + TKI (21.1)	CABO (43.9), other TKIs (22.8), ICI + TKI (21.1), LENVA + EVE (12.2)	54.4	45.6	20.0	57.8	6.4	24.9	8/low
Choueiri/2023 [28]	Phase 2/26.4	Intern.	52	100	ICI (54) or ICI + TKI (46)	Belzutifan + CABO	56	44	31	61	13.8	24.1	8/low
Dizman/2023 [8]	Retrospective/17.1	US	38	98.1	ICI + TKI (100)	PEMBRO + AXI	55	45	25	52.7	9.7	-	7/moderate
Vauchier/2022 [26]	Prospective/14.9	France	45	91	ICI (78), NIVO + IPI (11) or ICI + TKI (6)	ICI (85) or NIVO + IPI (15)	42	58	16	31	3.5	24	7/moderate
Lee/2022 [15]	Phase 1b-2/16.6	US	104	100	ICI	PEMBRO + LENV	39	61	62.5	29.8	12.2	Not reached	8/low
Tachibana/2022 [24]	Retrospective/6.7	Japan	27 *	76	ICI (96)	CABO	37	63	33	55	10.7 (not reached for nonRCC)	Not reached	6/high
Choueiri/2022 [6]	Phase 2/3.7	Internat.	46	100	ICI alone (track 2)	NIVO + IPI	21.7	76	17.4	41.3	3.7	23.8	6/low
Ornstein/2019 [18]	Phase 2/8.7	US	40	85	ICI (100)	AXI	28	72	45	45	8.8	-	7/low
Nadal/2016 [17]	Retrospective/7.8	US	68	100	ICI (70) or ICI + TKI (30)	AXI (67), PAZO (14), SUN (16), other (3), CABO (0)	25	75	27.9	42.6	6.4	16.9	6/low
Kwok/2023 [27]	Retrospective/NR	US	71	84.5	ICI (79), ICI + TKI (21)	LENVA + EVE	2.8	97.2	50	50	-	8.3 (2–3 lines)	6/high

*, n = 7 non-clear cell carcinoma; °, primary refractory; **, ICI alone or in combinations with other ICIs or experimental drugs; ^^, second line only; #, rabdoid or sarcomatoid differentiation; ##, second line only. Abbreviations: Atezo: Atezolizumab; Axi: Axitinib; Bev: Bevacizumab; Cabo: Cabozantinib; Clear cell RCC: clear cell renal cell carcinoma; com: combination; Eve: Everolimus; HDIL2: high-dose IL2; ICI: immune checkpoint inhibitor; Ipi: Ipilimumab; Lenva: Lenvatinib; mFU: median follow-up; mTORi: mTOR inhibitor; Nivo: Nivolumab; Pembro: Pembrolizumab; TKI: tyrosine kinase inhibitor. The proportion of patients receiving second-line therapy varied widely from 2.8% to 100%, with a median of 100%. One study utilized high-dose interleukin-2 (IL2) as the preferred treatment. The majority of the studies primarily concentrated on patients diagnosed with clear cell RCC, thus ensuring a consistent patient population throughout the studies. Each study included a sample size of 500 patients. First-line treatments were predominantly ICIs, either as monotherapy or in combination. In contrast, second-line treatments encompassed a wider range of TKIs that are commonly administered immediately or subsequent to a prior line of therapy, following disease progression on ICI therapies.

## Data Availability

All data are available from the corresponding author upon reasonable request.
